# Supplementary material for: Polyphenolic Compounds Inhibit Osteoclast Differentiation While Reducing Autophagy through Limiting ROS and the Mitochondrial Membrane Potential
Source: Biomolecules. 2022 Sep 1;12(9):1220. doi: 10.3390/biom12091220 (PMC9496366; doi:10.3390/biom12091220)
Supplement: Supplementary file 1 [file biomolecules-12-01220-s001.zip › biomolecules-1778534-supplementary.pdf]

Supplementary Figures

Polyphenolic Compounds Inhibit Osteoclast Differentiation by Reducing Autophagy through Limiting ROS and the Mitochondrial Membrane Potential

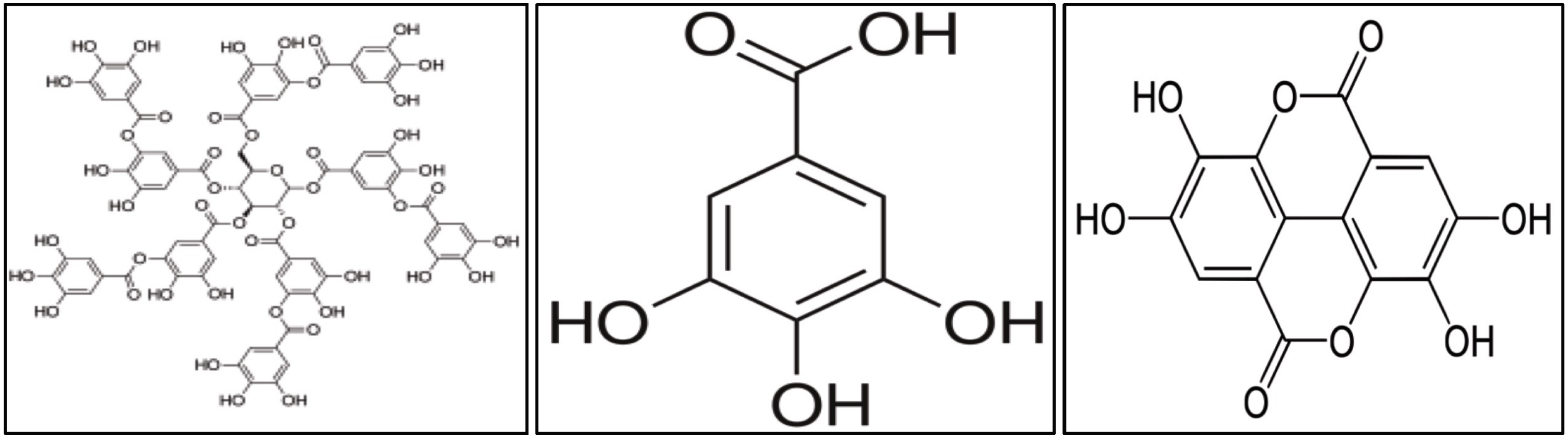

Tannic Acid (TA) **Gallic Acid (GA)** **Ellagic Acid (EA)**

Figure S1. Chemical structures of tannic, gallic and ellagic acids are shown.

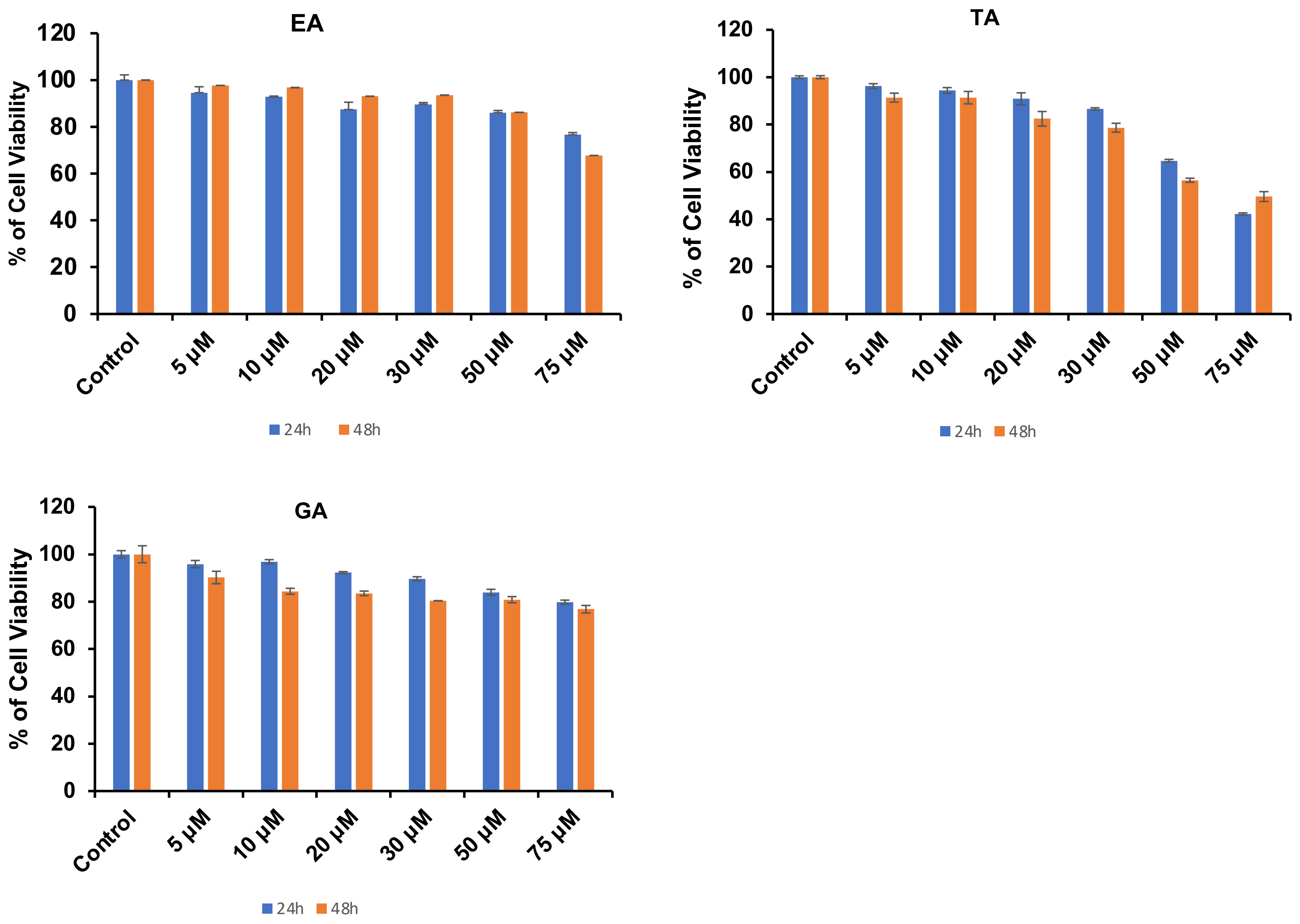

Figure S2. Dose-dependent effect of ellagic acid (EA), gallic acid (GA), and tannic acid (TA) on RAW264.7 cell viability are shown graphically.

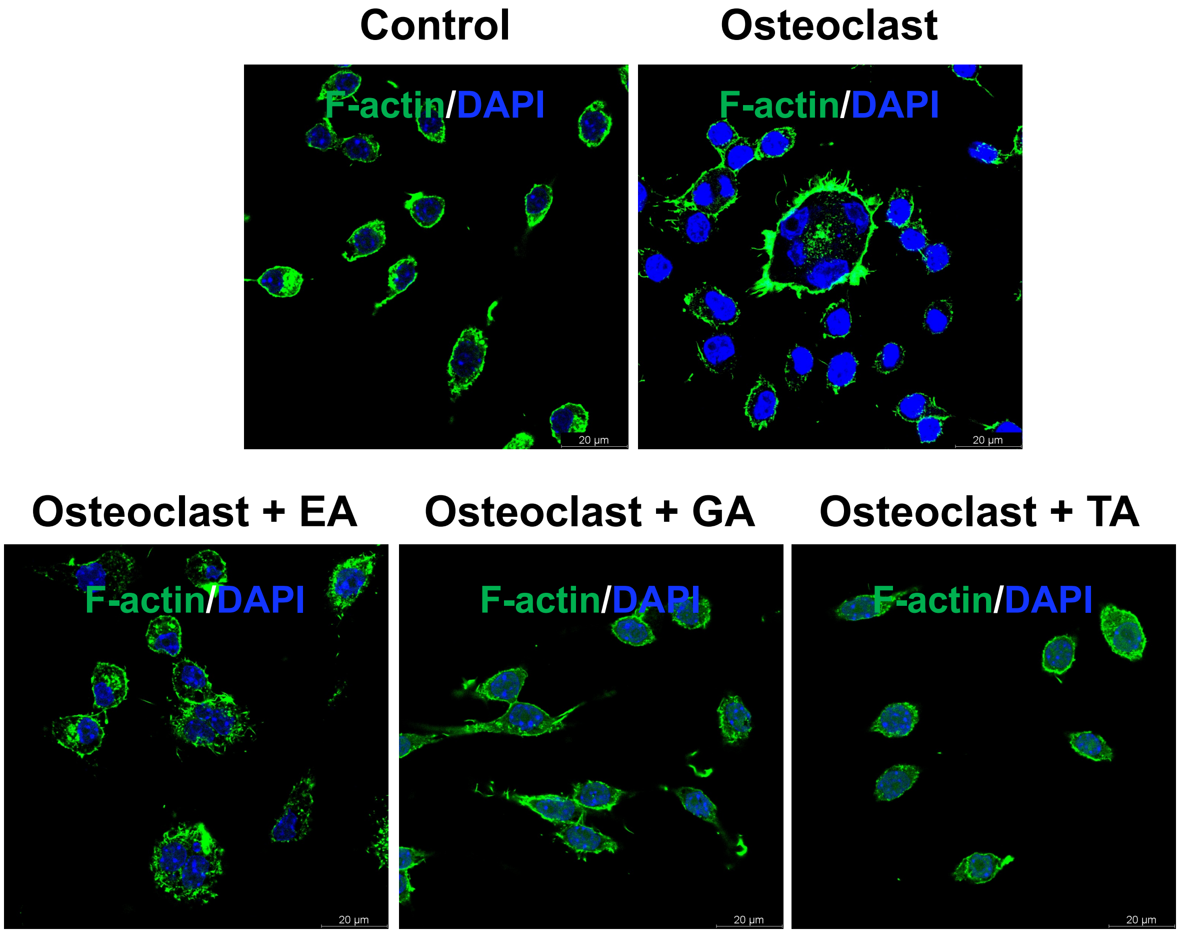

Figure S3. The effect of ellagic acid (EA), gallic acid (GA), and tannic acid (TA) on actin ring formation of osteoclasts. RAW264.7 cells (1x104 cells/well) were cultured on a coverslip inserted in a well of a 6-well plate and differentiated for 6 days in the presence or absence of polyphenolic compounds. After differentiation, cells were washed with ice-cold 1 x PBS and fixed with 4% PFA for 10 min. Next, cells were permeabilized with 0.1% Triton X-100 for 10 min at room temperature. Cells were then stained for 50 µg/mL FITC-conjugated phalloidin (#P5282; Sigma Aldrich, St Louis, USA) for 45 min at room temperature. Cells were then washed with 1 x PBS 3 times. The coverslips containing cells were then mounted on a slide using DAPI, and the borders were sealed with transparent nail varnish. Imaging was performed under a super-resolution confocal microscope (Leica Stellaris 8 STED, Ger-many), using a 100x objective, and images were analyzed using LAS X image analysis software. Each experiment was performed in triplicate.

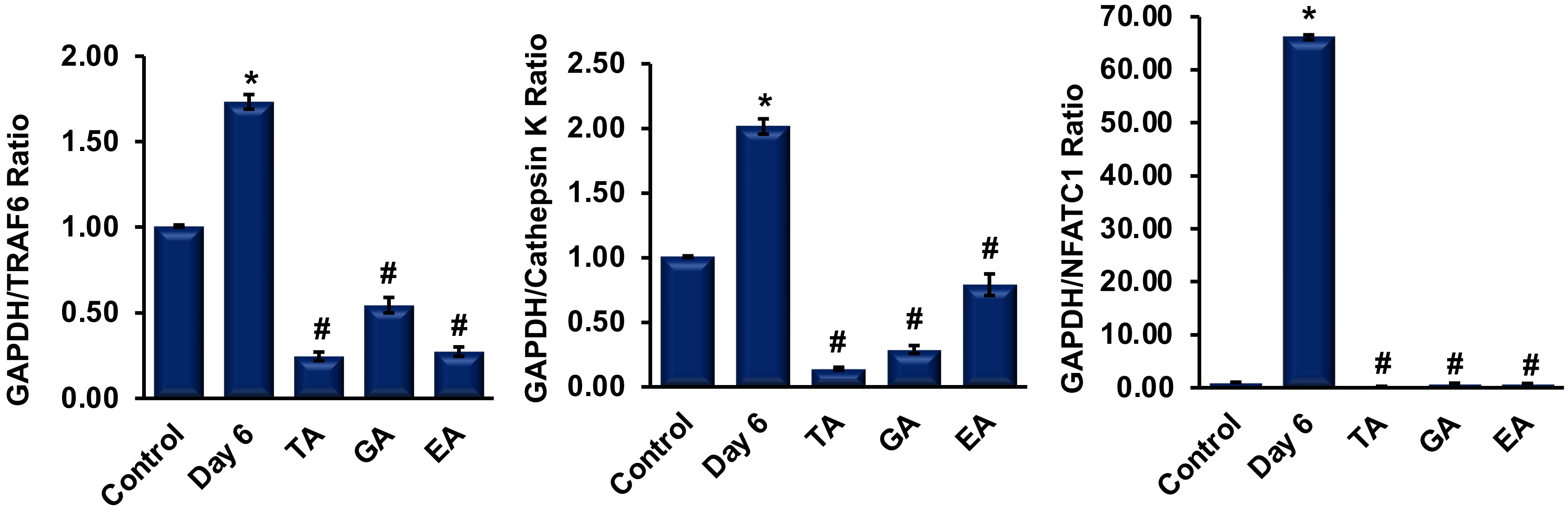

Figure S4. Bar graphs showing the quantification of TRAF6, Cathepsin K and NFATC1 compared to the loading control (GAPDH) of Fig. 4A. Star (*) indicates a statistical significance ($p<0.05$) when compared to controls with OC differentiated cells (Day 6); and hashtag (#) indicates a statistical significance ($p<0.05$) compared with differentiated cells respectively after addition of either TA, GA, or EA.

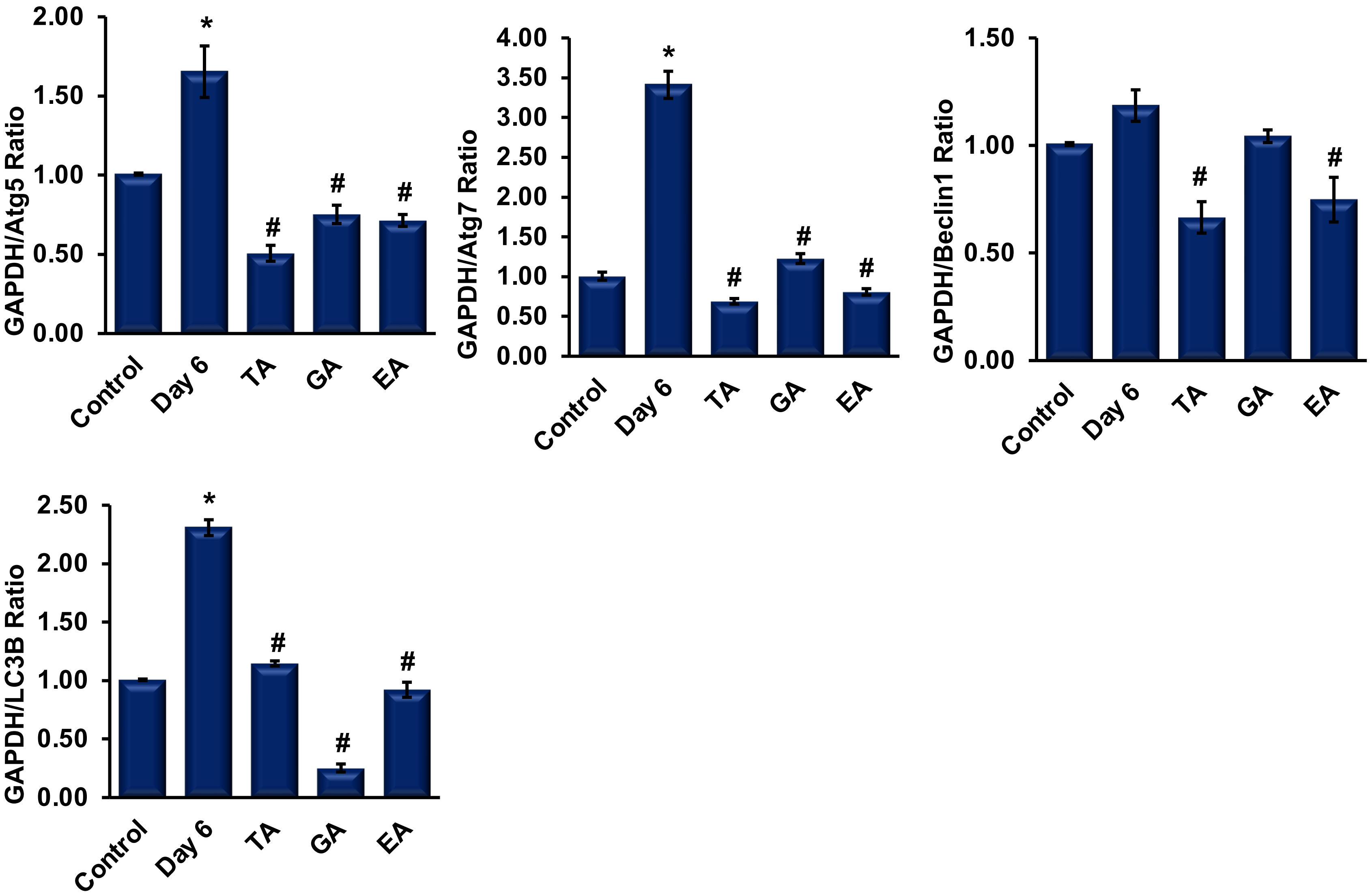

Figure S5. Bar graphs showing the quantification of autophagy markers such as ATG5, ATG7, Beclin1, and LC3B compared to the loading control (GAPDH) of Fig. 4B. Star (*) indicates a statistical significance ($p<0.05$) when compared to controls with OC differentiated cells (Day 6); and hashtag (#) indicates a statistical significance ($p<0.05$) compared with differentiated cells respectively after addition of either TA, GA, or EA.

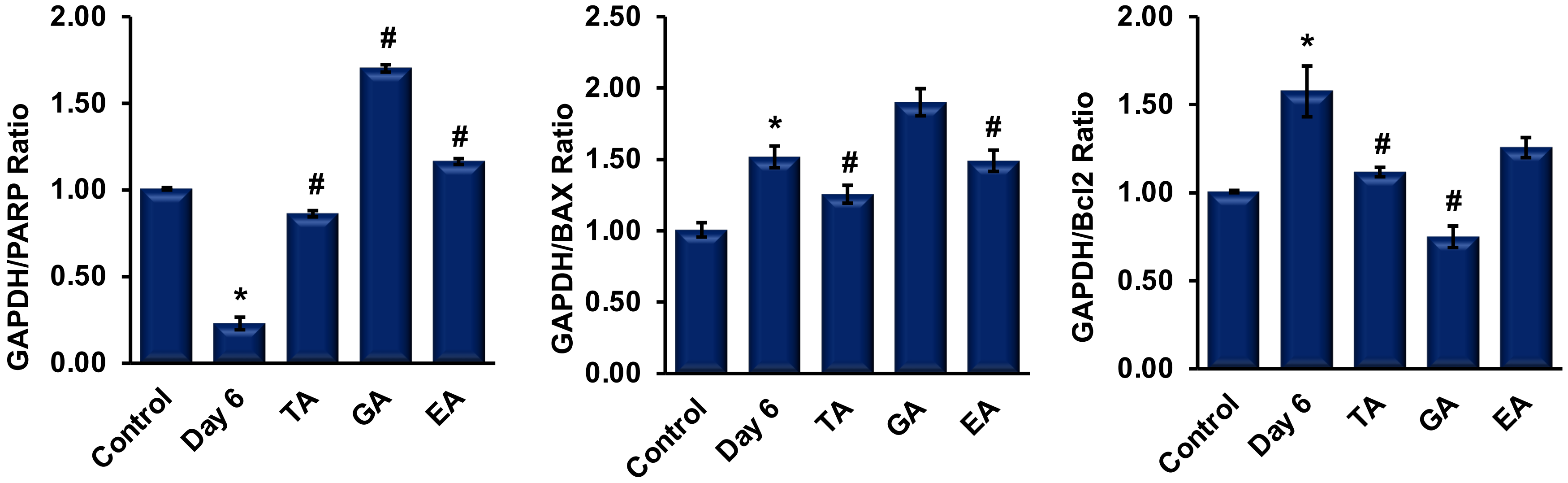

Figure S6. Bar graphs showing the quantification of apoptosis and cell survival related molecules such as PARP, BAX, and Bcl2 compared to the loading control (GAPDH) of Fig. 4C. Star (*) indicates a statistical significance ($p<0.05$) when compared to controls with OC differentiated cells (Day 6); and hashtag (#) indicates a statistical significance ($p<0.05$) compared with differentiated cells respectively after addition of either TA, GA, or EA.

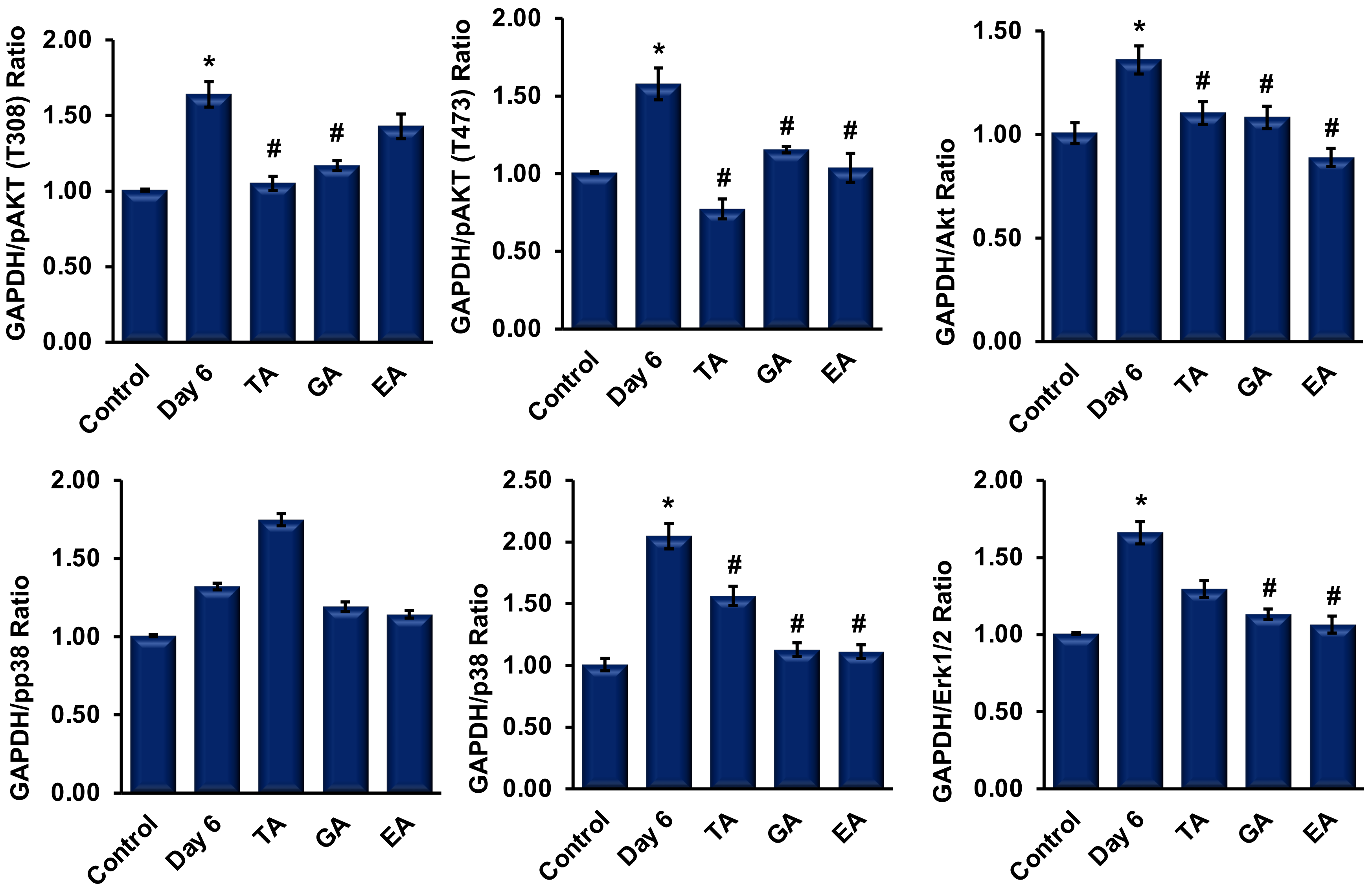

Figure S7. Bar graphs showing the quantification of pAKT (T308), pAKT (T473), Akt, pP38, p38,and ERK1/2 compared to the loading control (GAPDH) of Fig. 4D. Star (*) indicates a statistical significance ($p<0.05$) when compared to controls with OC differentiated cells (Day 6); and hashtag (#) indicates a statistical significance ($p<0.05$) compared with differentiated cells respectively after addition of either TA, GA, or EA.
